# Prostate apoptosis response-4 and tumor suppression: it’s not just about apoptosis anymore

**DOI:** 10.1038/s41419-020-03292-1

**Published:** 2021-01-07

**Authors:** Anees Rahman Cheratta, Faisal Thayyullathil, Siraj Pallichankandy, Karthikeyan Subburayan, Ameer Alakkal, Sehamuddin Galadari

**Affiliations:** Cell Death Signaling Laboratory, Division of Science, Experimental Research Building, New York University Abu Dhabi, PO Box 129188 Saadiyat Island Campus, Abu Dhabi, UAE

**Keywords:** Tumour-suppressor proteins, Apoptosis, Stress signalling, Target identification

## Abstract

The tumor suppressor prostate apoptosis response-4 (Par-4) has recently turned ‘twenty-five’. Beyond its indisputable role as an apoptosis inducer, an increasing and sometimes bewildering, new roles for Par-4 are being reported. These roles include its ability to regulate autophagy, senescence, and metastasis. This growing range of responses to Par-4 is reflected by our increasing understanding of the various mechanisms through which Par-4 can function. In this review, we summarize the existing knowledge on Par-4 tumor suppressive mechanisms, and discuss how the interaction of Par-4 with different regulators influence cell fate. This review also highlights the new secretory pathway that has emerged and the likely discussion on its clinical implications.

## Facts

- Par-4 is depleted, mutated, or inactivated in a myriad of cancers.
- Par-4 induces apoptosis specifically in some cancer cells, but not in normal or immortalized cells.
- Par-4 participates in ‘noncanonical’ tumor suppressive mechanism(s) such as autophagy, senescence, and metastasis inhibition.
- Par-4 is secreted by cancer cells as well as normal cells and the secreted Par-4 is able to induce apoptosis selectively in cancer cells

## Open questions

- What is the mechanistic basis fundamental to the ability of Par-4 to induce autophagy- and senescence-mediated tumor suppression?
- What is the importance of domains such as the P-loop domain, adenylyl cyclase domain, and the N-glycosylation site in Par-4 function?
- How does Par-4 mediate GRP78 translocation to the plasma membrane?
- Secreted Par-4 is able to efficiently prevent cancer development, how do we still develop cancer?

## Introduction

Prostate apoptosis response-4 (Par-4) is a therapeutically promising tumor suppressor protein encoded in humans by the *PAWR* gene. Par-4 was originally identified in rat prostate cancer cells undergoing apoptosis^1^. The most striking feature of Par-4 is that it’s ectopic expression or activation induces apoptosis specifically in some cancer cells but not in normal or immortalized cells^2,3^. Par-4 is ubiquitously expressed in tissues across different species^4^ and is found to be depleted, mutated, or inactivated in a myriad of cancers^5–11^. For instance, loss or mutation of genetic material around chromosome 12q21, where the human *PAWR* gene harbors^12^, is noted in Wilms’ tumorigenesis^12,13^ and in human male germ cell tumor development^14^. Diminished expression of Par-4 is evinced in the presence of oncogenic Ras, and restoration or overexpression of Par-4 induces apoptosis in these cells^15,16^. Given that Par-4 induces apoptosis, Par-4 knock-out mice spontaneously develop tumors in various organs, while transgenic mice overexpressing Par-4 were resistant to the growth of spontaneous or oncogene-inducible tumors^17,18^.

Besides the established role of Par-4 in restraining cancer, altered expression of Par-4 is noted in various non-cancerous ailments^19–24^. In this review, we focus only on the anticancer functions of Par-4. In fact, most of Par-4 functions have been considered in light of how Par-4 might induce apoptosis-mediated tumor suppression. However, recent studies are questioning whether Par-4 is truly such a “single-effect” protein? Other tumor suppressive functions of Par-4 that might be as important as apoptosis are being uncovered. Indeed, Par-4 has recently been found to regulate diverse anticancer mechanism(s), including metastasis, senescence, and autophagy. In this review, we shall summarize current knowledge of the cellular and molecular basis of Par-4-mediated tumor suppression; highlighting the recent seminal development in Par-4 research. Furthermore, we offer insight into the pathways that may contribute to non-apoptotic tumor suppression mediated by Par-4. This review will also focus on the therapeutic perspective of pharmacological reactivation of Par-4 and discuss issues that need to be addressed in order to bring Par-4 closer to the clinic.

## Dissecting the structural domains of Par-4

**Fig. 1:**
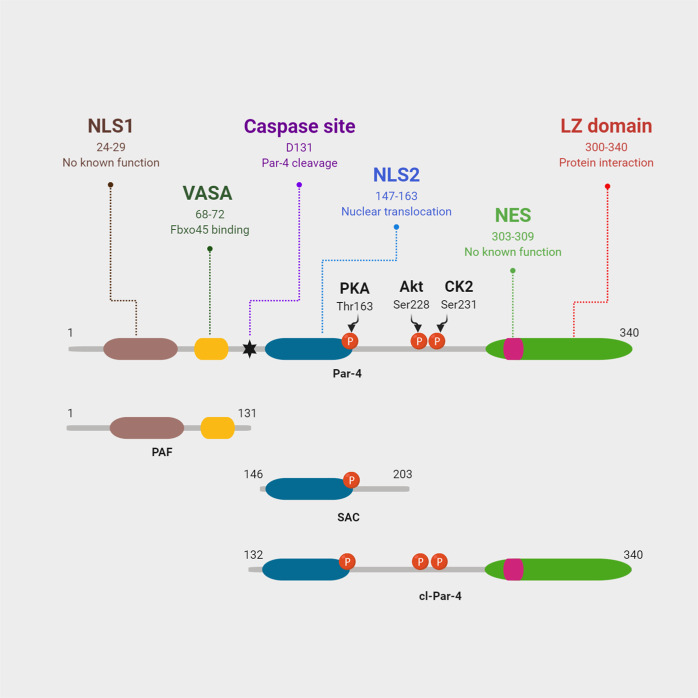
Schematic representation of human Par-4. The functional domains and some of the sites of post-translational modification are illustrated.

Human Par-4 is a ≈40 kDa protein containing 340 amino acids^25^. It contains several highly conserved well-delineated functional domains^2,26^ (Fig. 1). The presence of these motifs suggest that the Par-4 function may be closely regulated through protein–protein interaction, intercellular trafficking, and post-translational modification. Par-4 can exhibit its functions both in the cytoplasm and the nucleus, concordantly justified by the presence of two putative nuclear localization sequences (NLS1 and NLS2), and a nuclear export sequence (NES)^2,3,17^ (Fig. 1). Deletion of NLS2, but not NLS1, attenuates the nuclear translocation and apoptosis induction by Par-4. To date, NLS1 and NES have no known assigned Par-4 regulatory functions. At the C-terminal end, Par-4 has a leucine zipper domain (LZ domain)^25,27^ (Fig. 1) which functions as the primary recognition and binding site for various Par‐4 interacting proteins (Table 1). Par-4 deletion mutant lacking the LZ domain is still capable of inducing cell death. Therefore, it may well be assumed that the LZ domain is not indispensable for the cell death-inducing functions of Par-4^2^. Par-4 harbors a VASA domain (Fig. 1) which participates in its ubiquitylation and proteasomal degradation by Fbxo45 ubiquitin ligase^28,29^. Phosphorylation of Par-4 is classically regarded as an important regulatory mechanism of Par-4 function. Indeed, Par‐4 contains several conserved phosphorylation sites that are modified by kinases, such as protein kinase A (PKA)^30^, protein kinase B (PKB/Akt)^3,10^, and casein kinase-2 (CK-2)^31^ (Fig. 1). Depending on the site and the nature of the kinase, these modifications can inhibit or potentiate Par-4 activity.

**Table 1:** Par-4 interacting proteins.

Protein	Interaction	Domain	Ref
AATF/Che-1	Inhibits Par-4-mediated processing of β-amyloid precursor protein	LZ	^121^
Akt	Phosphorylates and sequesters Par-4 in the cytoplasm and blocks apoptosis	LZ, Thr163	^10,36^
Amida	Recruits Amida to actin cytoskeleton, leading to the induction of apoptosis	LZ	^122^
aPKC	Inhibits the activity of PKCζ, thereby inhibits PKCζ-mediated promotion of NF-κB activity	LZ	^40,41^
BACE1	Directly regulates amyloid precursor protein cleavage activity of BACE1	LZ	^19^
CK-2	Impairs the pro-apoptotic activity of Par-4	Ser231	^31^
D2DR	Facilitates Ca^2+^-mediated downregulation of D2DR efficacy and signaling	LZ	^123^
DAPK3	Relocates DAPK3 from the nucleus to the cytoplasm, particularly to actin filaments	LZ	^124^
E2F1	Indirectly controls the transcription of *Smac* gene through interacting with E2F1	LZ	^60^
Fbxo45	Promotes ubiquitylation and proteasomal degradation of Par-4	VASA	^28,29^
PKA	Promotes nuclear translocation of Par-4 and induces apoptosis	Ser228	^30,125^
SQSTM1/p62	Antagonizes Par-4-mediated inhibition of PKCζ by forming a ternary complex	LZ	^41^
THAP1	Potentiates both serum withdrawal- and TNFα-induced apoptosis	LZ	^126^
TOP1	Sequesters TOP1, attenuating its DNA relaxing catalytic activity	LZ	^127^
TRIM21	Down-regulates Par-4 and promotes cancer survival	NA	^128^
UACA	Prevents Par-4-mediated translocation of GRP78 from the ER to the cell surface	NA	^42^
Vimentin	Sequesters Par-4 preventing its secretion and extracellular function	NA	^74^
WT1	Repress WT1 and negatively modulates its transcription of Bcl-2, thereby induces apoptosis	LZ	^129,130^

*AATF/Che-1* apoptosis-antagonizing transcription factor, *aPKC* atypical protein kinase C, *BACE*1 beta-secretase 1, *D2DR* dopamine D2 receptor, *DAPK3* death-associated protein kinase 3, *E2F1* E2F transcription factor 1, *SQSTM1/p62* sequestosome 1, *THAP1* thanatos-associated domain-containing apoptosis-associated protein 1, *TOP1* topoisomerase 1, *TRIM21* Tripartite motif-containing protein 21, *WT1* Wilms’ tumor 1.

Structure-function analysis by El-Guendy et al. using a series of Par-4 mutants, identified a distinct and indispensable core domain, that when overexpressed, is sufficient to induce apoptosis selectively in cancer cells. Hence, the domain is termed as selective for apoptosis induction in cancer cells (SAC domain)^2^. This SAC domain induces apoptosis in cancer cells irrespective of their sensitivity or resistance to full-length Par-4-mediated apoptosis, yet does not induce apoptosis in normal cells^32^. The SAC domain contains the NLS2, which allows the nuclear entry of Par-4, and the PKA phosphorylation site, which is essential for Par-4 activation^2,30^. However, the SAC domain lacks Akt and CK-2 phosphorylation sites, which when phosphorylated, inhibit Par-4 activity^10,31^ (Fig. 1). This lack of negative regulation by Akt and CK-2 may very well explain why SAC induces apoptosis not only in Par-4 sensitive cells, but also in Par-4 resistant cells with elevated Akt activity^32^. Par-4 also bears a conserved caspase-specific cleavage site EEPD131G^33,34^ (Fig. 1) which is discussed later in this review.

## Par-4 interacting proteins: the nuts and bolts of Par-4 regulation

**Fig. 2:**
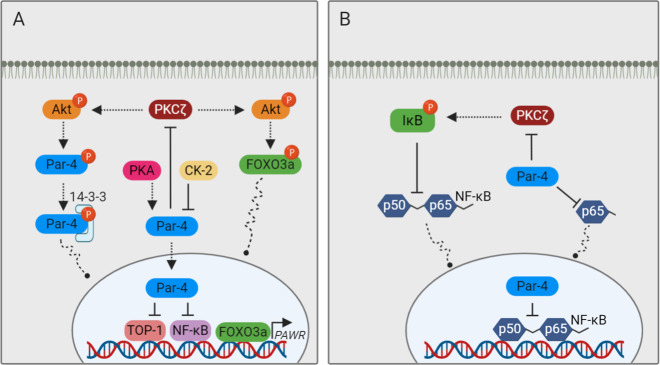
Par-4 interacting proteins. Some of the important positive and negative regulators of Par-4 functions are illustrated. See text for details.

Par-4 is a highly connected protein that can form a physical complex with many cytoplasmic and nuclear proteins, thereby, forming a multifaceted signaling network^25^. Some of the important direct Par-4 interacting proteins and their functions are depicted in Table 1. Nuclear translocation of Par-4 is essential for inhibition of nuclear factor kappa B (NF-κB)-dependent transcription activity, a key mechanism implicated in Par-4-mediated cytotoxicity. In fact Gurumurthy et al. have shown that PKA-mediated phosphorylation of Par-4 at Thr163 is critical for Par-4 nuclear translocation^30^ (Fig. 2A). Normal cells have relatively limited basal PKA activity when compared to cancer cells. This explains why normal cells are resistant to apoptosis by ectopic Par-4^30^. In contrast, Akt-mediated phosphorylation of Par-4 at Ser228 results in 14–3–3 chaperone-mediated sequestering of Par-4 in the cytoplasm and abrogation of apoptosis, even if Par-4 is phosphorylated on Thr163 by PKA^10,35,36^ (Fig. 2A). Akt can also regulate Par-4 function through Forkhead Box O3a (FOXO3a) transcription factor. Damrauer et al. and Das et al. have noted that Akt inhibition facilitates nuclear shuttling of FOXO3a, where it directly binds to the Par-4 promoter and activates Par-4 transcription^37,38^ (Fig. 2A). Overexpression of a constitutively active form of FOXO3a (lacking the Akt phosphorylation sites) induces Par-4 expression, whereas overexpression of Akt or silencing of FOXO3a activation inhibits the process^37,38^. Another serine/threonine kinase implicated in the regulation of Par-4 activity is CK-2. In humans, CK-2 phosphorylates Par-4 at Ser231 and impairs its pro-apoptotic activity^31^ (Fig. 2A).

In addition to kinases, Par-4 directly or indirectly interacts with an interesting array of pro-apoptotic and anti-apoptotic molecules and affects their function. One such molecule is the NF-κB transcription factor. NF-κB transcriptionally regulates genes generally involved in cell proliferation and survival. Par-4 modulates NF-κB function in a number of different ways. Recently, Par-4 has been shown to down-regulate NF-κB signaling through inhibition of p65 (also known as RelA; a subunit of NF-κB transcription complex) nuclear translocation and impedes leukemogenesis in an in vivo leukemia model^39^ (Fig. 2B). Additionally, Par-4 interacts with PKCζ and potentially inactivates the kinase by inducing a conformational change^40,41^, thereby, preventing the PKCζ-mediated phosphorylation of IκB, a step crucial for the nuclear trafficking of NF-κB^3^ (Fig. 2B). On the other hand, NF-κB has been shown to negatively regulate Par-4 activity via suppressing the Par-4 secretion^42^. Such an inverse correlation between Par-4 and NF-κB has also been reported in many other tumor suppression models^43–47^.

The effect of Par‐4 on PKCζ not only facilitates NF‐κB inhibition, but also inhibits Akt pro-survival kinase (Fig. 2A). Joshi et al. demonstrated that PKCζ directly interacts and phosphorylates Akt, which is significantly negated in Par‐4/PKCζ double knock-out cells. Simultaneously, PKCζ overexpression suppressed Par‐4’s inhibitory effect on Akt phosphorylation, indicating the existence of a Par‐4/PKCζ cassette that directly targets Akt^11,48^. Oncogenic Ras is another example of a pro-proliferative protein involved in an antagonistic relationship with Par-4^15,16,44,49^. Oncogenic Ras causes Par‐4 downregulation, while an increase in Par-4 expression, results in inhibition of Ras‐inducible cellular transformation^50^. However, the mechanistic underpinnings behind the Ras–Par-4 interaction is largely unclear. Pruitt et al. have observed that Ras-transformation induces MEK-dependent hypermethylation of Par-4 promoter. Treatment with the DNA methyltransferase inhibitor azadeoxycytidine restores Par-4 mRNA and protein levels, indicating that the mechanism for Ras-mediated downregulation of Par-4 is through promoter methylation^51^.

## Intracellular apoptosis: the canonical Par-4 function

**Fig. 3:**
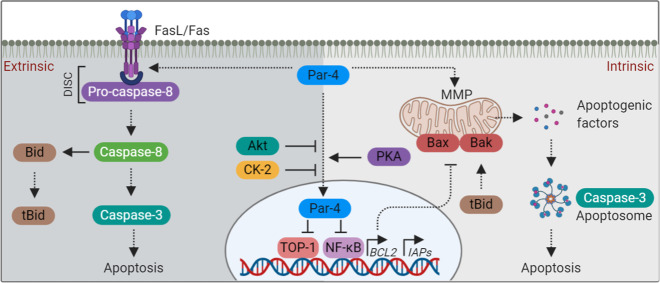
The canonical Par-4 functions. The extrinsic and intrinsic apoptotic pathway regulated by Par-4 is illustrated. The extrinsic cell death is initiated by the ligation of death-inducing ligands (TNFα, FasL, and TRAIL) to their cognate receptors (TNF receptor, Fas receptor, and death receptors-DR4/DR5). The ligation recruits adaptor proteins and pro-caspases, leading to the assembly of the DISC and the activation of caspase signaling cascade. The intrinsic pathway is characterized by permeabilization and depolarization of the mitochondrial membrane, leading to the release of apoptogenic factors such as cytochrome c, AIF, Smac/DIABLO, etc. Once released, these factors activate caspase signaling cascade and orchestrate the cell death process. Apoptosis is highly regulated by a variety of pro-apoptotic and anti-apoptotic proteins. The members of the Bcl-2 family of proteins are the central regulators of the intrinsic apoptotic pathway. Bcl-2 family proteins composed of both pro-apoptotic (e.g., Bad, Bid, Bax, Bak, Bcl-Xs, Bim, etc.) and anti-apoptotic (e.g., Bcl-2, Bcl-xL, Mcl-1, etc.) members. The balance between these two family members determines whether or not a cell will undergo apoptosis. tBid, truncated Bid; MMP, mitochondrial membrane permeabilization; DISC, death-inducing signaling complex.

Although ultimately converging, there are two distinct pathways (extrinsic and intrinsic) to apoptosis in mammalian cells^52,53^ (Fig. 3). The classical or canonical function of Par-4 is the induction of apoptosis through both the extrinsic and intrinsic pathways. In the context of extrinsic pathway, Par-4 appears to participate in cell death induced by various death ligands such as tumor necrosis factor-α (TNFα), Fas ligand (FasL), and TNF-related apoptosis-inducing ligand (TRAIL)^43,54–57^. For instance, Par-4 drives trafficking and activation of Fas receptor and FasL to the plasma membrane in hormone-independent prostate cancer cells^58^ (Fig. 3). Par-4-mediated Fas receptor mobilization, with a concomitant decrease in antiapoptotic cellular FLICE-like inhibitory protein (c-FLIP), leading to caspase activation has been observed in malignant lymphocytes^57^. Moreover, it has been shown that Par-4, along with TRAIL, increases death receptor-5 (DR5) expression and caspase activation, together with an enforced inactivation of anti-apoptotic XIAP and c-FLIP in renal cancer cells^43^.

In the context of the intrinsic apoptotic pathway, Boehrer et al. demonstrated that Par-4 expression in neoplastic lymphocytes, increases cleavage of caspase-8, activation of pro-apoptotic Bid and its translocation to the mitochondria, resulting in an augmentation of cytochrome c and AIF release into the cytoplasm^59^ (Fig. 3). Furthermore, Par-4 caused downregulation of B-cell lymphoma-2 (Bcl-2) and other inhibitors of apoptosis proteins (IAPs), such as cIAP1 and XIAP, leading to mitochondrial membrane permeabilization, caspase activation, and apoptosis^5^. Overexpression of par-4 in FasL-treated neoplastic lymphocytes promoted mitochondrial apoptosis *via* promoting Bid activation and AIF release^57^. Thus, Par-4 seems to impact at least three major intracellular apoptosis regulators, *viz*. Bcl-2, IAPs, and Bid. In addition to AIF and cytochrome c, Par-4 enhances Second mitochondria-derived activator of caspase/direct inhibitor of apoptosis-binding protein with low pI (Smac/DIABLO) release by transcriptionally modulating its expression through an indirect interaction between Par-4 and *Smac* promoter^60^. Par-4-dependent mitochondrial dysfunction and apoptosis was also reported in many other cancer and non-cancer models^20,61–64^. However, further investigation is required to unveil the complexity underlying the ability of Par-4 to regulate the release of apoptogenic factors, Bcl-2 family members, etc. The process of apoptosis is tightly regulated by a variety of pro-apoptotic and antiapoptotic proteins, lipids, and other molecules such as ROS. Par-4 function has not been studied in the context of these apoptosis regulators. Studies focused on aforesaid will outline a new step towards Par-4-dependent control of apoptosis.

## Caspase-mediated cleavage of Par-4: the cut that always bleeds

**Fig. 4:**
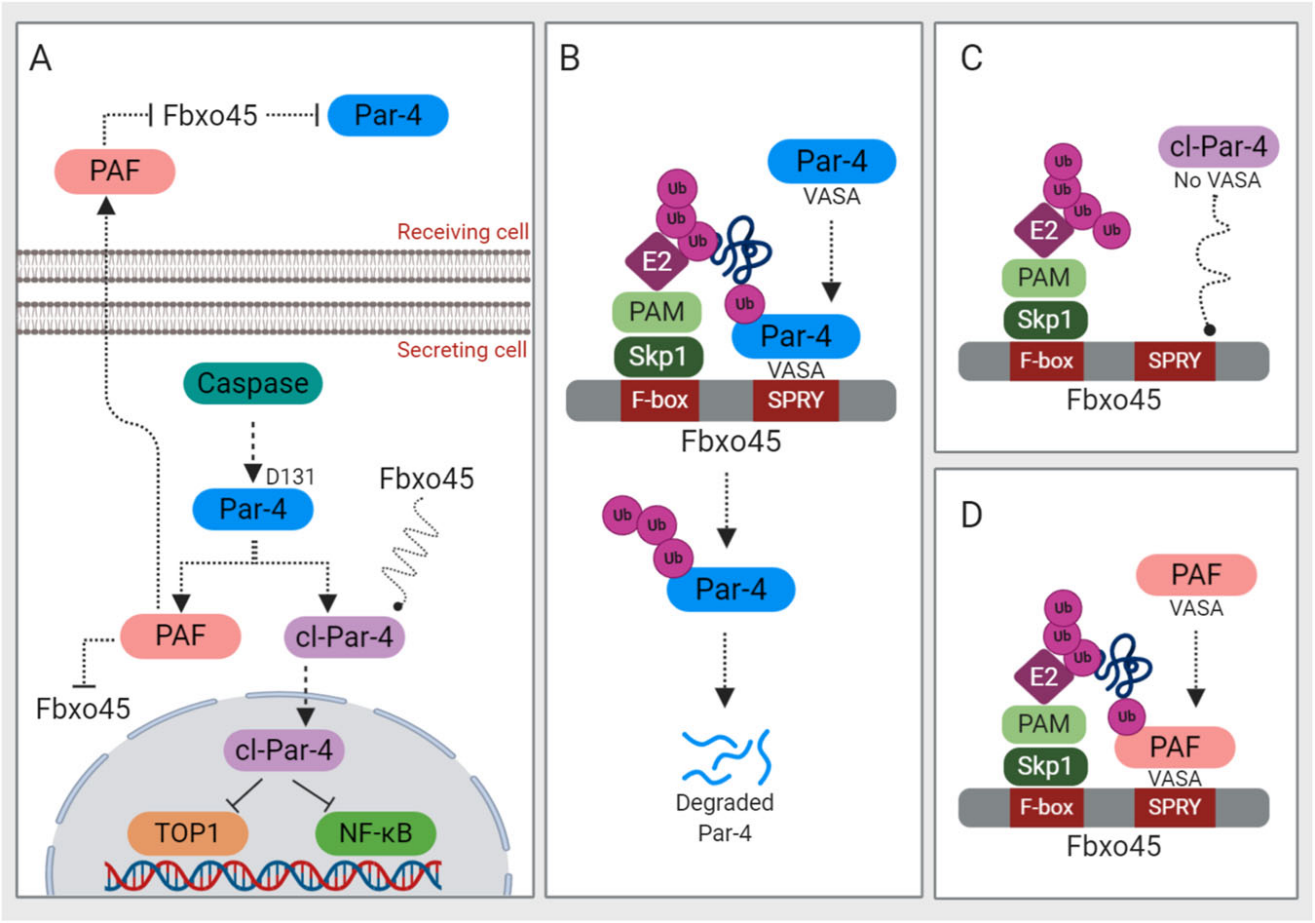
Caspase-mediated cleavage of Par-4. Caspase-dependent post-translational regulation of par-4 is illustrated. See text for details. Ub, ubiquitination.

Caspases are a unique family of cysteine proteases at the heart of networks that govern apoptosis. Caspase cleavage can result in gain-of-function, loss-of-function, or functional modification of the particular substrate^65^. Previously, Chaudhry et al. and our laboratory have demonstrated that caspases-3 cleaves in the N-terminal region of Par-4 at EEPD131↓G as a consequence of apoptosis, thereby generating two fragments: a 15 kDa amino-terminal fragment (Par-4 amino terminal fragment; PAF) and a 25 kDa carboxy-terminal fragment (cleaved Par-4; cl-Par-4)^33,34^ (Fig. 4A). The cl-Par-4 contains both the SAC domain and the LZ domain, while PAF contains the NLS1 and VASA region (Fig. 1). The cl-Par-4 containing NLS2 translocates into the nucleus and orchestrates its apoptotic function, whilst PAF through its VASA segment, rescues Fbxo45-mediated degradation of full-length Par-4^66^ (Fig. 4A, B). Cells transfected with D131A mutant Par-4 were completely resistant to cleavage during cisplatin treatment, and significantly reduced the number of apoptotic cells, suggesting that caspase-mediated cleavage confers gain-of-function on Par-4 protein^33^. Later, Treude et al. demonstrated that caspase-8 also generates cl-Par-4 in response to TNFα- and UV-induced apoptosis^67^. Indeed, cl-Par-4 was generated in response to a variety of anticancer agents effectively eliminating the cancer cells^33,34,67–70^.

Par-4 cleavage seems to enhance apoptosis *via* two inter-dependent mechanisms. First, cl-Par-4 is devoid of the VASA region where Fbxo45 binds and promotes ubiquitin-mediated degradation of Par-4 (Fig. 4C). Second, PAF (which is devoid of the functional Par-4 segment) generated from this process through its VASA region can interact with SPRY domain of Fbxo45, making Fbxo45 less available for the full-length Par-4 degradation (Fig. 4A, B, D). Taken together, we can conclude that PAF acts as a molecular decoy to rescue full-length Par-4 from Fbxo45-mediated degradation. It is particularly noteworthy that PAF selectively enters cancer cells, and not normal cells, hence, following its entry PAF induces apoptosis exclusively in cancer cells without affecting the normal cells (Fig. 4A). However, the precise mechanism conferring selective PAF entry in cancer cells still remains elusive. Interestingly, PAF induces apoptosis in both therapy-sensitive and therapy-resistant cancer cells^66^. For instance, taxane-resistant lung cancer cells, TRAIL-resistant prostate cancer cells, and PLX4720-resistant melanoma cells were all sensitive to apoptosis by PAF^66^. Further studies are warranted to know whether PAF could be used to overcome drug resistance in cancer treatment.

## Par-4 secretion: crossing the boundary

For many years, Par-4 investigations were predominantly focused on intracellular Par-4. Burikhanov et al. through a seminal paper, provided an entirely new dimension to the Par-4 paradigm. The authors demonstrated that Par-4 is secreted by both cancer and normal cells, however, the secreted Par-4 was able to induce apoptosis selectively in cancer cells. Furthermore, when ectopically introduced, both the full-length Par-4 and the SAC were intuitively secreted by normal and immortalized cells alike. Comparable to full-length Par-4, the secreted SAC also retained its pro-apoptotic potential against cancer cells, indicating that the SAC domain is critical for the apoptotic functions of secreted Par-4^71^. Surprisingly, Par-4 is also secreted in vivo into the serum or plasma of Par-4 transgenic mice and is available in the systemic circulation.

**Fig. 5:**
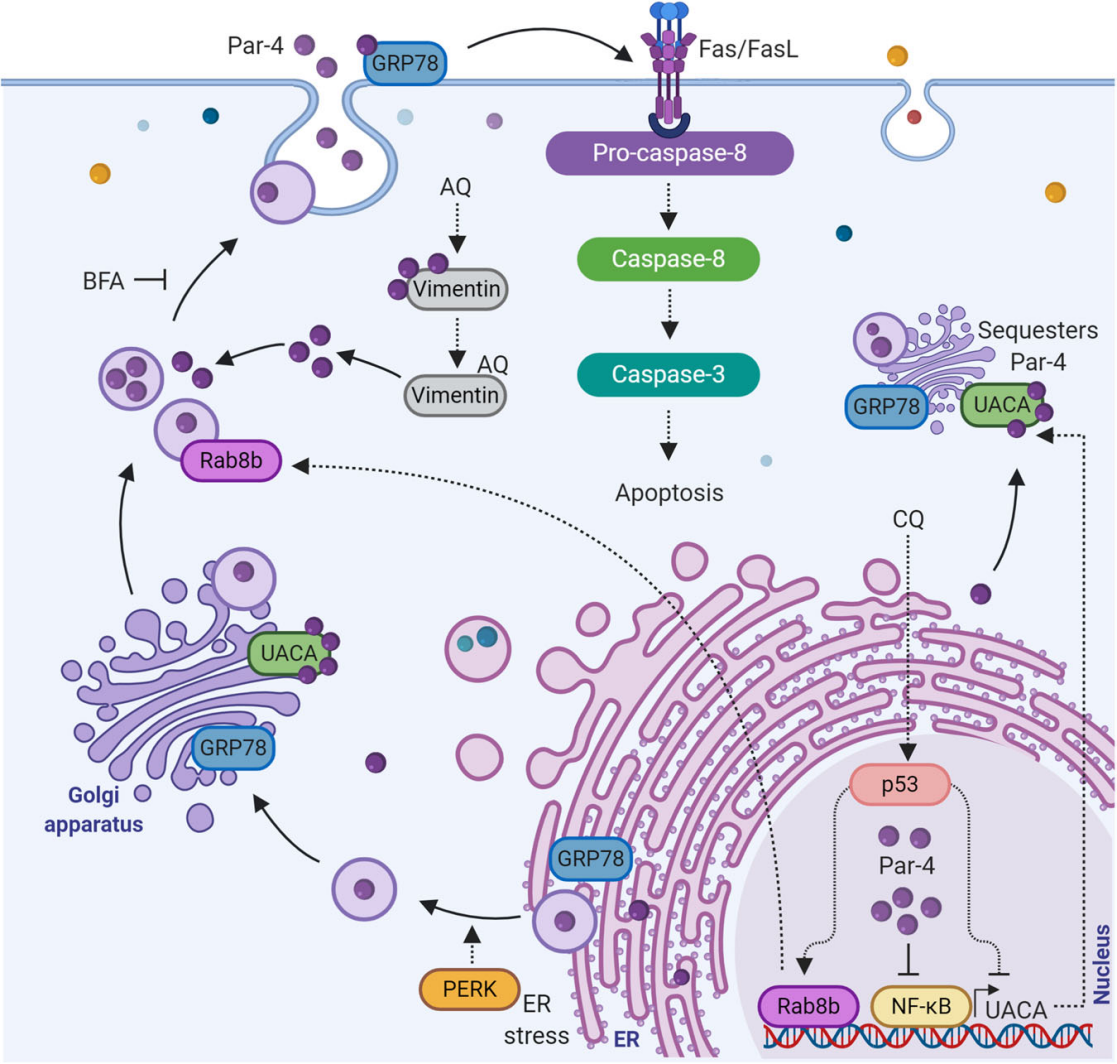
Par-4 secretion and secretagogues. Par-4 secretory pathway, important regulators of Par-4 secretion, and mechanism of Par-4 secretagogues are illustrated. See text for details.

Through a series of intricate and elegant experiments, Burikhanov et al. proved that (1) Par-4 secretion occurs through classical brefeldin A (BFA)-sensitive endoplasmic reticulum (ER)-Golgi secretory pathway, (2) ER chaperone protein glucose-regulated proteins-78 (GRP78) is a receptor for secreted Par-4 and SAC, and is essential for apoptosis by secreted Par-4 and SAC, (3) intracellular Par-4 facilitates GRP78 translocation to the cell surface in order to be available for apoptosis induction by secreted Par-4, and (4) secreted Par-4 activates apoptosis *via* caspase-8 and caspase-3 in a FADD-dependent manner^71^ (Fig. 5). The authors also noted that ER-stress seems to have a role in GRP78 translocation to the cell membrane and apoptosis mediated by secreted Par-4. PKR-like ER kinase (PERK) is an ER-stress activated protein known to promote apoptosis. When the cells were treated with recombinant Par-4, PERK phosphorylation was significantly increased and siRNA-mediated knock-down of PERK negated apoptosis by secreted Par-4^17,71^. In contrast to cancer cells, normal cells have a relatively lower expression of GRP78 at the cell surface. This may explain why cancer cells are more prone to the pro-apoptotic effect of secreted Par-4.

In a subsequent study, the same group identified uveal autoantigen with coiled-coil domains and ankyrin repeats (UACA) as a functional regulator of apoptosis induced by secreted Par-4^42^. The authors uncovered that UACA is a Par-4–binding protein, which sequesters Par-4 in the ER, preventing it from translocating GRP78 to the cell surface, and thereby, negatively regulating Par-4 secretion and the associated apoptosis (Fig. 5). Interestingly, UACA expression is positively regulated by NF-κB activity, and indeed, NF-κB-specific inhibitors caused suppression of UACA expression and promoted GRP78 translocation to the cell surface^42^ (Fig. 5). Since Par-4 can inhibit the NF-κB activity, this may prove to be yet another mechanism by which intracellular Par-4 can promote Par-4 secretion.

Later on, the same group came up with another interesting follow-up study where they demonstrated that the tumor suppressor p53 can directly bind to its consensus motif on *UACA*, and suppress the UACA expression in NF-kB-independent manner^72^ (Fig. 5). UACA is already known to sequester Par-4 and to prevent its secretion^42^. Therefore, inhibition of UACA by p53 activation and/or inhibition of NF-kB activity, resulted in elevated Par-4 secretion. The particular study also revealed that p53 activation in normal cells induces Par-4 secretion, leading to Par-4-dependent paracrine apoptosis in p53-deficient cells^72^. Similarly, studies using in vivo mice models have shown that normal cells in mice can be triggered to induce p53-dependent Par-4 secretion in systemic circulation. Serum secreted Par-4 is functionally active and it effectively induces ex vivo apoptosis in tumor cells, but not in normal cells^72^.

The mechanism behind Par-4 secretion was further elaborated by the same group, as they identified Rab8b (a member of the Rab family of GTPase) as a target of p53, essential for Par-4 transport from the Golgi to the plasma membrane *via* the classical secretory pathway. The authors elegantly proved that p53 directly binds to the promoter region of *Rab8b*, up-regulates Rab8b expression, and promotes Par-4 secretion in response to chloroquine (CQ), a recognized Par-4 secretagogue^73^ (Fig. 5). Furthermore, Par-4 can also physically interact with vimentin, a cytoskeletal intermediate filament protein. Another class of Par-4 secretagogue, arylquins (AQ), can enhance Par-4 secretion by binding with vimentin, displacing Par-4 from vimentin for secretion (Fig. 5), and efficiently inducing paracrine apoptosis of tumor cells^74^.

## Par-4 in autophagy: apoptosis is not enough

A growing body of work suggests that Par-4 also controls additional ‘noncanonical’ tumor suppressive mechanisms. Autophagic death is a degradation response whereby cellular proteins and organelles are engulfed, digested, and recycled to sustain cellular metabolism^75,76^. Recent evidence suggests that Par-4 can contribute to autophagy. Wang et al., reported simultaneous induction of apoptosis and autophagy by Par‐4 in hypopharyngeal carcinoma cells^77^. We have shown that curcumin induces ROS-dependent Par-4 generation, leading to autophagic cell death in human malignant glioma^78^. Interestingly, Par‐4 overexpression alone was sufficient to induce autophagic cell death. Extracellular treatment of H_2_O_2_ also induced Par‐4 accumulation and autophagic cell death, indicating that ROS generation is, indeed, an important regulator of Par-4-mediated autophagy^78^. In a follow-up study, we identified the tumor suppressor p53-dependent Bcl-2/adenovirus E1B 19 kDa interacting protein 3 (BNIP3) generation as a downstream target of Par-4-mediated autophagic cell death following curcumin treatment. Interestingly, the Par-4-p53-BNIP3 axis was also activated in response to other well-established autophagy inducers, including ceramide and arsenic trioxide, indicating that autophagy induction may very well be a significant Par-4-mediated tumor suppression mechanism^79^.

Resistance of cancer cells to chemotherapy is a significant clinical concern. Many cancers such as malignant glioma are capable of evading apoptosis^80^. For this reason, autophagy has received increasing scientific attention as an alternative cell death pathway. In this context, resolving the molecular mechanism behind Par-4-dependent regulation of autophagic cell death is of high significance. Further studies are warranted to know (1) how Par-4 is activated during autophagy induced by various anticancer agents? (2) what are the structural domains of Par-4 critical for its autophagic function and whether SAC domain is sufficient for autophagy? (3) is secreted Par-4 capable of inducing autophagy-mediated tumor suppression? (4) what determines if cells die by apoptosis or autophagy during Par-4 activation and whether this is influenced by Par-4 subcellular localization? Hopefully answers to these questions will be addressed in future studies.

## Par-4 in senescence: another layer of Par-4 function

Cellular senescence is an irreversible cell cycle arrest, the induction of which, is a promising strategy for the treatment and prevention of cancer^81^. p53 is the most crucial cell death protein which lies at the heart of senescence-mediated tumor suppression^82^. Although the Par-4 and p53 connection has been documented in several studies^72,73,83^, the specific role of Par-4 in this has never been fully explored. Previously, we have shown that Par-4-dependent p53 upregulation plays a critical role in thymoquinone-induced cellular senescence in human malignant glioma^84^. Notably, overexpression of Par-4 itself increased senescence in glioma cells, whereas, shRNA- or siRNA-mediated inhibition of Par-4 significantly negated the thymoquinone-induced senescence, hence, underscoring the prime role of Par-4. Furthermore, we observed that induction of Par-4 results in the activation of the p53/p21 pathway with concomitant inhibition of cell cycle progression in the G1 phase^84^. Similarly, Du et al. have demonstrated that Par-4 plays an important role in senescence of mouse cardiac fibroblasts. Ectopic expression of Par-4 promoted senescence, whereas, silencing of Par-4 repressed senescence when cells were cultured in serum-free medium or treated with H_2_O_2_^85^. However, studies on the role of Par-4 in senescence is only starting to evolve and further research is warranted to facilitate a better understanding of the mechanism involved.

## Par-4 in metastasis: navigating further

The dissemination of cancer cells from primary tumors and their subsequent colonization into distant tissues, referred to as metastasis, contributes to over 90% of cancer-associated deaths^86^. Invasion and angiogenesis are crucial prerequisite events to be followed by tumor cells on their path to metastasize. For invasion to happen, immortalized tumor cells have to acquire epithelial-to-mesenchymal transition (EMT), a trans‐differentiation process that allows cells to attain mesenchymal properties, such as mobility or capability to detach from surrounding cells^87^. Interestingly, Par-4 was shown to play a crucial role in the control of EMT. For instance, Fernandez-Marcos et al., noted that concomitant Par-4 ablation and PTEN-heterozygosity lead to invasive prostate carcinoma in mice^45^. Likewise, systemic elevation of Par-4 was shown to inhibit the growth of LLC1-derived metastatic lung tumor nodules in syngeneic mice^18^. Furthermore, ectopic expression of Par-4 was shown to inhibit the formation of lung nodules in a tail vein metastatic model and also reversed EMT in BXPC-3/CDDP cells^88,89^.

**Fig. 6:**
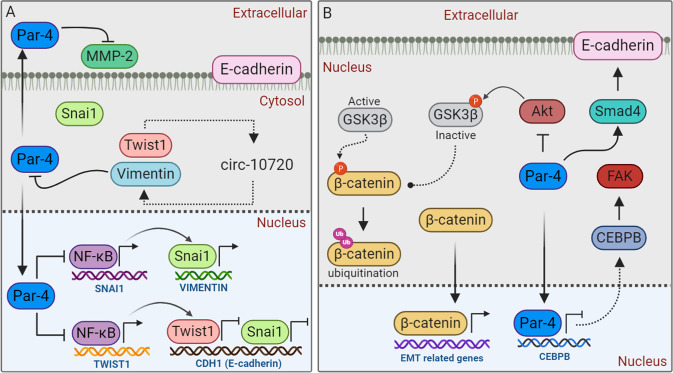
Schematic representation of Par-4 role in metastasis. Proposed mechanism of Par-4-dependent inhibition of metastasis are illustrated. See text for details.

Recently, Katoch et al. have demonstrated that Par-4 actively participates in directing various events in inhibiting EMT or facilitating mesenchymal-epithelial transition (MET; the reverse process to EMT) in metastatic pancreatic cancer cells^90^. Firstly, Par-4 significantly inhibits mesenchymal markers such as vimentin and Twist-related protein 1 (Twist‐1), while promoting epithelial marker such as E‐cadherin^90^. The mechanism as to how Par-4 regulates these markers are not explored in detail. However, it is hypothesized that Par‐4-mediated inhibition of NF‐κB might have a negative impact on the transcription factor snail homolog 1 (Snai1) and Twist‐1. Snai1 is known to positively regulate vimentin and negatively regulate E‐cadherin^90–92^, whereas, Twist‐1 can transcriptionally repress E‐cadherin promoter activity and promotes vimentin expression by increasing circ-10720 levels, which can absorb miRNAs that target vimentin^90,93–95^ (Fig. 6A). Importantly, vimentin was shown to be a direct Par-4 target, sequestering Par-4 and inhibiting its secretion^74^ (Fig. 6A). A sharp amplification of E‐cadherin expression was also observed by Amin et al., in GFP‐Par‐4 transfected cells^96^. Consistent with this report, Suman et al. have noted that oral administration of withaferin A induces Par-4 accumulation and up-regulates E-cadherin, while down-regulating mesenchymal markers such as β-catenin and vimentin in prostate cancer model^97^ (Fig. 6A, B). Secondly, Par-4 augments ALK2/Smad4 signaling which is crucial for the Par‐4‐mediated maintenance of E‐cadherin. Although, Smad4 has also been implicated in the induction of EMT^98,99^, several previous reports have shown that Smad4 can induce E‐cadherin accumulation or inhibit its reduction^100–102^ (Fig. 6B).

In an alternative mechanism, it has been reported that Par-4 can bind to the CCAAT enhancer binding protein beta (CEBPB) promoter and inhibit its transcription (Fig. 6B). CEBPB is a transcriptional regulator of focal adhesion kinase (FAK), a cytoplasmic non-receptor protein tyrosine kinase that serves an important role in cytoskeletal remodeling and EMT. In this particular study, Du et al. have demonstrated that miR-17–3p microRNA negatively regulates Par-4, leading to the upregulation of proteins like CEBPB, FAK, N-cadherin, vimentin, and the downregulation of E-cadherin^85^.

Burikhanov et al. tested the antimetastatic potential of Par-4 secretagogue CQ, whereby, CQ was found to induce robust Par-4 secretion and prevented the spread of EO771 mouse mammary tumor cells to the lungs in Par-4+/+ mice, but not in Par-4-deficient mice^73^. The presence of Par-4 antibody expressively negated the antimetastatic features, indicating that Par-4 secretion is critical for CQ-inducible inhibition of metastasis^73^. Recently, Nayak et al. noted that Indolylkojyl methane analogue (IKM5) induces the expression of Par-4, leading to Par-4-dependent tampering of GRP78-tissue inhibitor of metalloproteases 1 (TIMP-1) complex. IKM5 also promotes the nuclear localization of Par-4 (Perhaps due to GRP78 suppression), leading to diminished expression of Par-4 downstream target NF-kB, thereby, negatively regulating NF-kB-mediated pro-EMT activities in invasive cancer cells^103^ (Fig. 6A).

Another possible mechanism by which Par-4 participates in cell migration/metastasis is *via* modulating β-catenin. It has been reported that induction of intracellular Par-4 abrogates EMT and invasion in prostate and breast cancer cells through modulation of oncogenic β‐catenin^96^. Specifically, intracellular Par-4 (by suppressing the Akt/PKB) induces GSK3β activation, which in turn phosphorylates β‐catenin and prevents its nuclear accumulation (Fig. 6B), a common and early event during neoplastic progression and metastasis^96,104^. In another report, Rah et al. have demonstrated that extracellular Par‐4, secreted by a classical BFA-sensitive pathway, inhibits invasion and angiogenesis in human cervical cancer and prostate cancer cells *via* negatively regulating matrix metalloproteinases-2 (MMP‐2), an endopeptidase responsible for the proteolytic degradation of extracellular matrix (ECM) components^105^. How extracellularly secreted Par-4 modulates the MMP-2 activity will be an interesting topic to be explored in future studies. Taken together, Par‐4 can be exploited as a potential therapeutic target for the discovery of small molecule compounds to control aggressive metastatic cancer.

## Par-4 in cancer therapeutics: its brighter on the other side

For decades, the mainstay and goal of clinical oncology has been revolved around development of therapies that effectively kill cancer cells. Classical chemotherapeutic agents imprecisely target both cancer and non-cancerous cells, resulting in detrimental and often life-threatening toxicities in patients. Therefore, over the past two decades, significant efforts have been channeled to identify targeted therapies to specifically eradicate cancer cells, while sparing the patient’s normal cells. For this reason, Par-4 offers an attractive target for developing anticancer therapy. Given that cancer cells are generally more susceptible than normal cells to the pro-apoptotic effect of Par-4, therapeutic strategies targeting Par-4 are expected to have minimal, if any, side effects. Furthermore, Par-4 can induce apoptosis in the absence of p53 and PTEN function, both of which are lost or non-functional in most of the cancers^83,106^. Hence, activating PAR-4 could be a promising strategy for the treatment of cancers which carries non-functional p53 or PTEN. So how might Par-4 be activated? Several strategies have been developed in the past few years to reactivate or potentiate Par-4.

The detailed analysis of the Par-4 interacting proteins has allowed the validation of a number of suitable targets that permits pharmaceutical control of Par-4 levels in cells. In many tumors, Par-4 activity is held dormant by the overexpression of its negative regulators, *viz;* Akt, Bcl-2, oncogenic Ras amongst others^15,16,36,38,107,108^. Therefore, one obvious goal is to try and re-establish the cytotoxic functions of Par-4 in cancer cells by targeting these molecules. Multiple inhibitors against these molecules are now in various stages of clinical trial, albeit they are not explored for their ability to induce Par-4 reactivation^109–111^. For instance, suppression of Akt activation by the PI3K-inhibitor (LY294002), Akt expression by RNA-interference, or Akt function by dominant-negative Akt caused apoptosis in cancer cells via Par-4-dependent manner^10^. Similarly, Akt inhibitor ISC-4 caused Par-4 activation and reduced tumor growth in a colon cancer nude mouse model^112^. Azmi et al. demonstrated that nonpeptidic small-molecule inhibitors of Bcl-2 family proteins (apogossypolone and TW-37) induced Par-4-dependent inhibition of cell growth and induction of apoptosis in pancreatic cancer cells and inhibited tumor growth in xenograft animal model^113^. According to recent data, ~19 different Bcl-2 inhibitors (antibodies, small molecule drugs, and antisense oligonucleotides) are undergoing preclinical or clinical studies for the treatment of different cancers^110^. It will be interesting to evaluate whether some of these agents can induce Par-4. Furthermore, transient overexpression of oncogenic kras in wild-type-kras BxPC-3 cells significantly downregulated the endogenous Par-4 protein levels and conferred accelerated growth. The above-described studies suggest that small molecules that disrupt Par-4 interactions with its negative regulators could be utilized to reactivate Par-4 in cancer therapy.

There has been a wealth of data, both in vitro and in vivo, demonstrating the efficacy of Par-4 in orchestrating cell death in many cancer types. For instance, advanced prostate cancer containing a mixture of androgen-responsive and androgen-refractory cells, are resistant to apoptosis by androgen ablation, the mainstay of prostate cancer therapy. Srinivasan et al. have demonstrated that withaferin A when combined with anti-androgens induces apoptosis in both androgen-responsive and androgen-refractory prostate cancer cells^83^. Activation of Par-4 also reverts the inherently observed chemoresistance in pancreatic cancer towards gemcitabine and cisplatin, two commonly used agents for the treatment of pancreatic cancer^107^. Taken together, Par-4 is a possible target candidate that would benefit prostate and pancreatic cancer therapy. Overexpression of Par-4 alone was sufficient to induce tumor suppression in various cancer models. A single intra-tumoral injection of an adenoviral-Par-4 construct resulted in significant apoptosis in over 80% of tumor cells^58^. Kline et al., have used nanoliposomes to effectively deliver Par-4 plasmid into tumors, whereby, nanoliposomes facilitate Par-4 expression and increase apoptosis in response to 5-FU in vitro. Moreover, tumors in mice treated with Par-4 nanoliposomes were more susceptible to 5-FU treatment in vivo^114^. Very recently, Kim et al. developed a novel long-lasting form of Par-4 (Par-4Ex) by using *E. coli* expression system. Par-4Ex improved the biological half-life in mice by ~7-fold and effectively suppressed the metastatic tumor growth^115^. The evidence that Par-4 is important for cell death in many cancer cell types is mounting. Par-4 is increased in response to agents that are in the clinic, *viz;* 5-FU^47,114^, doxorubicin^5^, etoposide^70^, and radiotherapy^46^. Indeed, a large variety of anticancer agents have been identified that can effectively activate Par-4 and eliminate cancer cells (Table 2).

**Table 2:** Various stimuli inducing Par-4 and their mode of cell death.

Stimulus	Model system	Response	Ref
3-azidowithaferin A	HeLa and PC3	↓Invasion and angiogenesis	^105^
3-azidowithaferin A	MCF7 and DU145	↓EMT and invasion	^96^
All-trans-retinoid acid	LNln3	↑Apoptosis	^130^
Arsenic trioxide	U87MG and U118MG	↑Autophagy	^79^
Ceramide	U87MG and U118MG	↑Autophagy	^79^
COX inhibitors	HCA-7	↑Apoptosis	^131^
Cranberry	DU145	↑Apoptosis	^132^
Curcumin	U87MG and U118MG	↑Autophagy	^78,79^
Docetaxel	MCF7	↑Apoptosis	^133^
Doxorubicin	Jurkat	↑Apoptosis	^5^
Imatinib	DOHH-2 and WSU-NHL	↑Apoptosis	^134^
ISC-4	Nude Mouse	↑Tumor reduction	^112^
Lapatinib	BT-474	Improve survival	^135^
Ouabain	PC3	↑Apoptosis	^136^
Parasporin-2Aa1	PC3	↑Apoptosis	^137^
Raloxifene	EPN	↑Apoptosis	^138^
Sanguinarine	PC3 and DU145	↑Apoptosis	^68^
Tamoxifen	HNGC-2	↑Apoptosis	^64^
Thymoquinone	U87MG	↑Senescence	^84^
Withaferin A	PC3	↑Apoptosis	^83^

Part of the tumor resistance to the apoptotic effect of wild-type Par-4 involves its capability to get phosphorylated and inactivated by Akt and CK-2. Remarkably, the SAC domain lacking these phosphorylation sites, demonstrated moderately high anticancer efficacy compared to wild-type Par-4, therefore, making it a highly attractive anticancer target^2^. For instance, SAC transgenic mice demonstrated resistance to spontaneous, as well as, oncogene-induced tumor growth with no significant interference with the development and life span of the animal^88^. Simultaneously, SAC- (and also Par-4-) transgenic mice expressing systemic SAC/Par-4 protein resisted the non-autochthonous tumor growth^18^. Sarkar et al. have engineered a plant-derived SAC-Par-4-GFP protein which is biologically active and able to reduce growth of the prostate cancer cell lines^116^. Qin et al. designed a secretory protein by joining a secretory signal peptide NT4(Si) to the Par-4 SAC-HA2TAT peptide gene sequence [NT4(Si)-Par-4 SAC-HA2TAT], and demonstrated that this fusion protein suppressed cell growth and induced rapid cell death in HepG2 cells^117^. Recently, Zhang et al. produced native SAC in *E. coli* using a small ubiquitin-related modifier (SUMO) fusion system. Not only the fusion system improved the solubility of SAC, but also it induced apoptosis in SKOV-3 ovarian cancer cells^118^.

The fundamental discovery that Par-4 can be secreted (by both normal and cancer cells), and that the secreted Par-4 exhibits a bystander apoptotic effect (only in cancer cells) has produced a wonderful ‘hot spot’ to target Par-4 for cancer-selective therapeutics. By virtue of this bystander effect, tumor cells that are distally located from Par-4 overexpressing cells are inhibited as a result of increased Par-4 protein^119^. The clinical importance of this finding is highlighted by the observation that SAC transgenic protein is released in the blood stream of SAC transgenic mice. The secreted Par-4/SAC was stable and systemically active for an extended period of time (over 4 months), and was able to effectively suppress tumor growth and inhibit metastasis in mice^18^. Moreover, the serum secreted Par-4 effectively induced ex vivo apoptosis in tumor cells, but not in normal cells^18,72^, suggestive of its considerable promise in terms of designing novel strategies for anticancer therapeutics.

Owing to the profound clinical impact exhibited by the secreted Par-4, secretagogues that augment Par-4 release could be more interesting. Large-scale chemical screening for Par-4 secretagogues has led to the identification of Arylquin-1 and FDA-approved anti-malarial drugs, CQ and hydroxychloroquine (HCQ), as robust inducers of Par-4 secretion^73,74^. Arylquin-1 caused Par-4 secretion from normal cells and triggered efficient apoptosis in cancer cells only when they were co-cultured^74^. CQ caused elevated systemic Par-4 secretion in mice and induced paracrine apoptosis in distally located cancer cells^73^. Collectively, these findings suggest that Par-4 secretagogues have tremendous therapeutic potential and may become a useful therapy for effectively alleviating cancer. However, further extensive studies are required to evaluate the efficacy of Par-4 secretagogues in human subjects. Expectedly, such clinical studies are currently in progress (NCT03015324 and NCT02232243). Results of a phase I clinical trial with 2-week neoadjuvant oral administration of HCQ in patients with surgically removable early stage solid tumors showed an elevation in plasma Par-4 levels with no significant toxicities. The resected tumors from the HCQ-treated patients exhibited TUNEL-positivity correlating with tumor apoptosis^120^.

## Summary and future direction

**Fig. 7:**
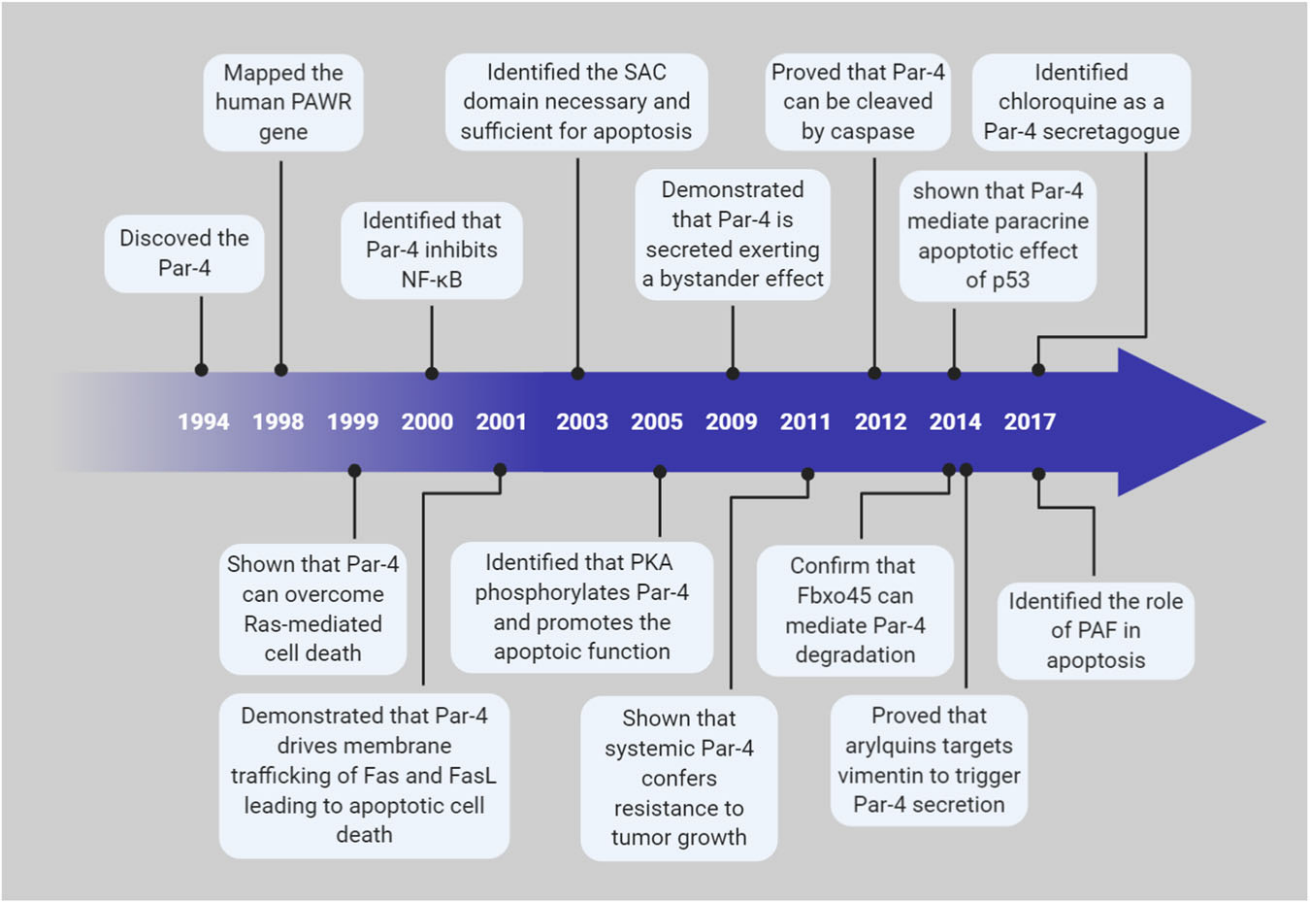
Major milestones in Par-4 research. Timeline highlighting crucial discoveries that provided insights into Par-4 functions and regulation.

Over the years, detailed characterization of Par-4 protein interaction and post-translational modifications have produced fundamental insights into the dynamics of Par-4-mediated tumor suppressive mechanism(s) (Fig. 7). However, despite this intensive effort, there is still much left to learn. It is now becoming clear that Par-4 has a much broader role than inducing apoptosis. Indeed, it contributes to other tumor suppressive pathways including autophagic cell death, senescence, and metastasis. The fundamental mechanistic basis for the ability of Par-4 to induce these varied biological responses remains largely unclear, and hence, ripe for investigation and elucidation. Par-4 has several uncharacterized, yet interesting, structural domains needing to be investigated as discussed elsewhere^3^. These structural domains represent some of the intriguing challenges that need to be addressed in future investigations.

Par-4 exerts its bystander effects through membrane trafficking and binding to GRP78. Par-4 lacks a typical signal peptide for its transport into the ER. So how would Par-4 mediate GRP78 translocation? Characterization of the biochemical details of the interaction between GRP78 and Par-4, and in general, elucidation of the secretion pathway, warrants further investigation. Despite its secretion from normal cells, endogenous Par-4 does not induce apoptosis on its own. One proposed mechanism for this involves decreased GRP78 expression in normal cells as opposed to cancer cells. However, this requires more validation. Perhaps more importantly, when secreted Par-4 is able to efficiently prevent cancer development, but then how do we still develop cancer? Perhaps the secreted Par-4 is kept in a dormant state in cancer, or is it possible that Par-4 secretion is diminished in cancer when compared to normal conditions? These open questions concerning the regulation of Par-4 must be addressed in future studies.

Although there is still much to learn, it seems clear that manipulating the Par-4 pathway will undoubtedly bring considerable therapeutic benefits. There are few Par-4 secretagogues currently being tested that could prove substantially effective in the clinic at some point in the future. The results thus far are highly encouraging and we have only just begun the exploration for Par-4 secretagogues. It is reasonable to hope that Par‐4 strategically could be a powerful tool for developing anticancer therapeutics.

## References

[1] Sells SF (1994). Commonality of the gene programs induced by effectors of apoptosis in androgen-dependent and -independent prostate cells. Cell Growth Differ..

[2] El-Guendy N, Zhao Y, Gurumurthy S, Burikhanov R, Rangnekar VM (2003). Identification of a unique core domain of par-4 sufficient for selective apoptosis induction in cancer cells. Mol. Cell Biol..

[3] Hebbar N, Wang C, Rangnekar VM (2012). Mechanisms of apoptosis by the tumor suppressor Par-4. J. Cell Physiol..

[4] El-Guendy N, Rangnekar VM (2003). Apoptosis by Par-4 in cancer and neurodegenerative diseases. Exp. Cell Res..

[5] Boehrer S (2002). In lymphatic cells par-4 sensitizes to apoptosis by down-regulating bcl-2 and promoting disruption of mitochondrial membrane potential and caspase activation. Cancer Res.

[6] Cook J (1999). Decreased expression of the pro-apoptotic protein Par-4 in renal cell carcinoma. Oncogene.

[7] Moreno-Bueno G (2007). Inactivation of the candidate tumor suppressor par-4 in endometrial cancer. Cancer Res..

[8] Kogel D (2001). Dlk/ZIP kinase-induced apoptosis in human medulloblastoma cells: requirement of the mitochondrial apoptosis pathway. Br. J. Cancer.

[9] Nagai MA (2010). Down-regulation of the candidate tumor suppressor gene PAR-4 is associated with poor prognosis in breast cancer. Int J. Oncol..

[10] Goswami A (2005). Binding and phosphorylation of par-4 by akt is essential for cancer cell survival. Mol. Cell.

[11] Joshi J (2008). Par-4 inhibits Akt and suppresses Ras-induced lung tumorigenesis. EMBO J..

[12] Johnstone RW, Tommerup N, Hansen C, Vissing H, Shi Y (1998). Mapping of the human PAWR (par-4) gene to chromosome 12q21. Genomics.

[13] Austruy E (1995). Characterization of regions of chromosomes 12 and 16 involved in nephroblastoma tumorigenesis. Genes Chromosomes Cancer.

[14] Murty VV (1996). Physical mapping of a commonly deleted region, the site of a candidate tumor suppressor gene, at 12q22 in human male germ cell tumors. Genomics.

[15] Barradas M, Monjas A, Diaz-Meco MT, Serrano M, Moscat J (1999). The downregulation of the pro-apoptotic protein Par-4 is critical for Ras-induced survival and tumor progression. EMBO J..

[16] Qiu SG, Krishnan S, el-Guendy N, Rangnekar VM (1999). Negative regulation of Par-4 by oncogenic Ras is essential for cellular transformation. Oncogene.

[17] Shrestha-Bhattarai T, Rangnekar VM (2010). Cancer-selective apoptotic effects of extracellular and intracellular Par-4. Oncogene.

[18] Zhao Y (2011). Systemic Par-4 inhibits non-autochthonous tumor growth. Cancer Biol. Ther..

[19] Xie J, Guo Q (2005). PAR-4 is involved in regulation of beta-secretase cleavage of the Alzheimer amyloid precursor protein. J. Biol. Chem..

[20] QiNan W (2016). Par-4/NF-kappaB Mediates the Apoptosis of Islet beta Cells Induced by Glucolipotoxicity. J. Diabetes Res..

[21] Qinan W, Ling Z, Bing C (2014). PAR-4: a possible new target for age-related disease. Expert Opin. Ther. Targets.

[22] Deng JT, Bhaidani S, Sutherland C, MacDonald JA, Walsh MP (2019). Rho-associated kinase and zipper-interacting protein kinase, but not myosin light chain kinase, are involved in the regulation of myosin phosphorylation in serum-stimulated human arterial smooth muscle cells. PLoS ONE.

[23] Vetterkind S (2010). Par-4: a new activator of myosin phosphatase. Mol. Biol. Cell.

[24] Wang G (2012). Astrocytes secrete exosomes enriched with proapoptotic ceramide and prostate apoptosis response 4 (PAR-4): potential mechanism of apoptosis induction in Alzheimer disease (AD). J. Biol. Chem..

[25] Libich DS (2009). Intrinsic disorder and coiled-coil formation in prostate apoptosis response factor 4. FEBS J..

[26] Tiruttani Subhramanyam UK, Kubicek J, Eidhoff UB, Labahn J (2017). Structural basis for the regulatory interactions of proapoptotic Par-4. Cell Death Differ..

[27] Sells SF (1997). Expression and function of the leucine zipper protein Par-4 in apoptosis. Mol. Cell Biol..

[28] Chen X (2014). Fbxo45-mediated degradation of the tumor-suppressor Par-4 regulates cancer cell survival. Cell Death Differ..

[29] Wang Z, Wei W (2014). Fbxo45 joins the ‘Par-4’ty in controlling apoptosis of cancer cells. Cell Death Differ..

[30] Gurumurthy S, Goswami A, Vasudevan KM, Rangnekar VM (2005). Phosphorylation of Par-4 by protein kinase A is critical for apoptosis. Mol. Cell Biol..

[31] de Thonel A (2014). Regulation of the proapoptotic functions of prostate apoptosis response-4 (Par-4) by casein kinase 2 in prostate cancer cells. Cell Death Dis..

[32] Zhao Y, Rangnekar VM (2008). Apoptosis and tumor resistance conferred by Par-4. Cancer Biol. Ther..

[33] Chaudhry P, Singh M, Parent S, Asselin E (2012). Prostate apoptosis response 4 (Par-4), a novel substrate of caspase-3 during apoptosis activation. Mol. Cell Biol..

[34] Thayyullathil F (2013). Caspase-3 mediated release of SAC domain containing fragment from Par-4 is necessary for the sphingosine-induced apoptosis in Jurkat cells. J. Mol. Signal.

[35] Kline CL, Irby RB (2011). The pro-apoptotic protein prostate apoptosis response protein-4 (Par-4) can be activated in colon cancer cells by treatment with Src inhibitor and 5-FU. Apoptosis.

[36] Zhuang D (2012). TMZ-induced PrPc/par-4 interaction promotes the survival of human glioma cells. Int J. Cancer.

[37] Damrauer JS (2018). Foxo-dependent Par-4 Upregulation prevents long-term survival of residual cells following PI3K-Akt inhibition. Mol. Cancer Res..

[38] Das TP, Suman S, Alatassi H, Ankem MK, Damodaran C (2016). Inhibition of AKT promotes FOXO3a-dependent apoptosis in prostate cancer. Cell Death Dis..

[39] Greene JT (2019). Par-4 overexpression impedes leukemogenesis in the Emicro-TCL1 leukemia model through downregulation of NF-kappaB signaling. Blood Adv..

[40] Diaz-Meco MT (1996). The product of par-4, a gene induced during apoptosis, interacts selectively with the atypical isoforms of protein kinase C. Cell.

[41] Chang S, Kim JH, Shin J (2002). p62 forms a ternary complex with PKCzeta and PAR-4 and antagonizes PAR-4-induced PKCzeta inhibition. FEBS Lett..

[42] Burikhanov R (2013). Novel mechanism of apoptosis resistance in cancer mediated by extracellular PAR-4. Cancer Res..

[43] Lee TJ (2010). Overexpression of Par-4 sensitizes TRAIL-induced apoptosis via inactivation of NF-kappaB and Akt signaling pathways in renal cancer cells. J. Cell Biochem.

[44] Diaz-Meco MT, Lallena MJ, Monjas A, Frutos S, Moscat J (1999). Inactivation of the inhibitory kappaB protein kinase/nuclear factor kappaB pathway by Par-4 expression potentiates tumor necrosis factor alpha-induced apoptosis. J. Biol. Chem..

[45] Fernandez-Marcos PJ (2009). Simultaneous inactivation of Par-4 and PTEN in vivo leads to synergistic NF-kappaB activation and invasive prostate carcinoma. Proc. Natl Acad. Sci. USA.

[46] Chendil D, Das A, Dey S, Mohiuddin M, Ahmed MM (2002). Par-4, a pro-apoptotic gene, inhibits radiation-induced NF kappa B activity and Bcl-2 expression leading to induction of radiosensitivity in human prostate cancer cells PC-3. Cancer Biol. Ther..

[47] Wang BD (2010). Prostate apoptosis response protein 4 sensitizes human colon cancer cells to chemotherapeutic 5-FU through mediation of an NF kappaB and microRNA network. Mol. Cancer.

[48] Diaz-Meco MT, Moscat J (2008). Akt regulation and lung cancer: a novel role and mechanism of action for the tumor suppressor Par-4. Cell Cycle.

[49] Ahmed MM (2008). Downregulation of PAR-4, a pro-apoptotic gene, in pancreatic tumors harboring K-ras mutation. Int J. Cancer.

[50] Vasudevan KM, Ranganathan P, Rangnekar VM (2006). Regulation of Par-4 by oncogenic Ras. Methods Enzymol..

[51] Pruitt K (2005). Ras-mediated loss of the pro-apoptotic response protein Par-4 is mediated by DNA hypermethylation through Raf-independent and Raf-dependent signaling cascades in epithelial cells. J. Biol. Chem..

[52] Tang D, Kang R, Berghe TV, Vandenabeele P, Kroemer G (2019). The molecular machinery of regulated cell death. Cell Res..

[53] Taylor RC, Cullen SP, Martin SJ (2008). Apoptosis: controlled demolition at the cellular level. Nat. Rev. Mol. Cell Biol..

[54] Shareef MM (2007). Role of tumor necrosis factor-alpha and TRAIL in high-dose radiation-induced bystander signaling in lung adenocarcinoma. Cancer Res..

[55] Notaro A (2014). Involvement of PAR-4 in cannabinoid-dependent sensitization of osteosarcoma cells to TRAIL-induced apoptosis. Int J. Biol. Sci..

[56] Shelke, G. V. et al. TNF-alpha and IFN-gamma together up-regulates Par-4 expression and induce apoptosis in human neuroblastomas. *Biomedicines* **6**, 4 (2017). 10.3390/biomedicines6010004 PMC5874661 29278364

[57] Bergmann M (2004). Prostate apoptosis response gene-4 sensitizes neoplastic lymphocytes to CD95-induced apoptosis. Ann. Hematol..

[58] Chakraborty M, Qiu SG, Vasudevan KM, Rangnekar VM (2001). Par-4 drives trafficking and activation of Fas and Fasl to induce prostate cancer cell apoptosis and tumor regression. Cancer Res..

[59] Boehrer S (2004). Upon drug-induced apoptosis expression of prostate-apoptosis-response-gene-4 promotes cleavage of caspase-8, bid and mitochondrial release of cytochrome c. Hematology.

[60] Lu C (2008). Multimolecular complex of Par-4 and E2F1 binding to Smac promoter contributes to glutamate-induced apoptosis in human- bone mesenchymal stem cells. Nucleic Acids Res..

[61] Duan W, Rangnekar VM, Mattson MP (1999). Prostate apoptosis response-4 production in synaptic compartments following apoptotic and excitotoxic insults: evidence for a pivotal role in mitochondrial dysfunction and neuronal degeneration. J. Neurochem..

[62] Chan SL, Tammariello SP, Estus S, Mattson MP (1999). Prostate apoptosis response-4 mediates trophic factor withdrawal-induced apoptosis of hippocampal neurons: actions prior to mitochondrial dysfunction and caspase activation. J. Neurochem.

[63] Xie J, Awad KS, Guo Q (2005). RNAi knockdown of Par-4 inhibits neurosynaptic degeneration in ALS-linked mice. J. Neurochem.

[64] Jagtap JC (2014). Expression and regulation of prostate apoptosis response-4 (Par-4) in human glioma stem cells in drug-induced apoptosis. PLoS ONE.

[65] Julien O, Wells JA (2017). Caspases and their substrates. Cell Death Differ..

[66] Hebbar N (2017). A naturally generated decoy of the prostate apoptosis response-4 protein overcomes therapy resistance in tumors. Cancer Res.

[67] Treude F (2014). Caspase-8-mediated PAR-4 cleavage is required for TNFalpha-induced apoptosis. Oncotarget.

[68] Rahman A, Pallichankandy S, Thayyullathil F, Galadari S (2019). Critical role of H_2_O_2_ in mediating sanguinarine-induced apoptosis in prostate cancer cells via facilitating ceramide generation, ERK1/2 phosphorylation, and Par-4 cleavage. Free Radic. Biol. Med.

[69] Brasseur K (2016). Post-translational regulation of the cleaved fragment of Par-4 in ovarian and endometrial cancer cells. Oncotarget.

[70] Guo H, Treude F, Kramer OH, Luscher B, Hartkamp J (2019). PAR-4 overcomes chemo-resistance in breast cancer cells by antagonizing cIAP1. Sci. Rep..

[71] Burikhanov R (2009). The tumor suppressor Par-4 activates an extrinsic pathway for apoptosis. Cell.

[72] Burikhanov R (2014). Paracrine apoptotic effect of p53 mediated by tumor suppressor Par-4. Cell Rep..

[73] Burikhanov R (2017). Chloroquine-Inducible Par-4 Secretion Is Essential for Tumor Cell Apoptosis and Inhibition of Metastasis. Cell Rep..

[74] Burikhanov R (2014). Arylquins target vimentin to trigger Par-4 secretion for tumor cell apoptosis. Nat. Chem. Biol..

[75] Levy JMM, Towers CG, Thorburn A (2017). Targeting autophagy in cancer. Nat. Rev. Cancer.

[76] Doherty J, Baehrecke EH (2018). Life, death and autophagy. Nat. Cell Biol..

[77] Wang LJ (2014). Concomitant induction of apoptosis and autophagy by prostate apoptosis response-4 in hypopharyngeal carcinoma cells. Am. J. Pathol..

[78] Thayyullathil F, Rahman A, Pallichankandy S, Patel M, Galadari S (2014). ROS-dependent prostate apoptosis response-4 (Par-4) up-regulation and ceramide generation are the prime signaling events associated with curcumin-induced autophagic cell death in human malignant glioma. FEBS Open Bio.

[79] Thayyullathil, F. et al. Par-4 regulates autophagic cell death in human cancer cells via upregulating p53 and BNIP3. *Biochim. Biophys. Acta. Mol. Cell. Res.* **1867**, 118692 (2020). 10.1016/j.bbamcr.2020.118692 32135176

[80] Kaza N, Kohli L, Roth KA (2012). Autophagy in brain tumors: a new target for therapeutic intervention. Brain Pathol..

[81] Campisi J (2001). Cellular senescence as a tumor-suppressor mechanism. Trends Cell Biol..

[82] Rufini A, Tucci P, Celardo I, Melino G (2013). Senescence and aging: the critical roles of p53. Oncogene.

[83] Srinivasan S, Ranga RS, Burikhanov R, Han SS, Chendil D (2007). Par-4-dependent apoptosis by the dietary compound withaferin A in prostate cancer cells. Cancer Res.

[84] Subburayan K, Thayyullathil F, Pallichankandy S, Rahman A, Galadari S (2018). Par-4-dependent p53 up-regulation plays a critical role in thymoquinone-induced cellular senescence in human malignant glioma cells. Cancer Lett..

[85] Du WW (2015). The microRNA miR-17-3p inhibits mouse cardiac fibroblast senescence by targeting Par4. J. Cell Sci..

[86] Lambert AW, Pattabiraman DR, Weinberg RA (2017). Emerging biological principles of metastasis. Cell.

[87] Yeung KT, Yang J (2017). Epithelial-mesenchymal transition in tumor metastasis. Mol. Oncol..

[88] Zhao Y (2007). Cancer resistance in transgenic mice expressing the SAC module of Par-4. Cancer Res..

[89] Tan J (2014). Par-4 downregulation confers cisplatin resistance in pancreatic cancer cells via PI3K/Akt pathway-dependent EMT. Toxicol. Lett..

[90] Katoch A (2018). Dual role of Par-4 in abrogation of EMT and switching on Mesenchymal to Epithelial Transition (MET) in metastatic pancreatic cancer cells. Mol. Carcinog..

[91] Kaufhold S, Bonavida B (2014). Central role of Snail1 in the regulation of EMT and resistance in cancer: a target for therapeutic intervention. J. Exp. Clin. Cancer Res.

[92] Cano A (2000). The transcription factor snail controls epithelial-mesenchymal transitions by repressing E-cadherin expression. Nat. Cell Biol..

[93] Meng J (2018). Twist1 Regulates Vimentin through Cul2 Circular RNA to Promote EMT in Hepatocellular Carcinoma. Cancer Res.

[94] Tang H (2016). AKT-ions with a TWIST between EMT and MET. Oncotarget.

[95] Vesuna F, van Diest P, Chen JH, Raman V (2008). Twist is a transcriptional repressor of E-cadherin gene expression in breast cancer. Biochem Biophys. Res. Commun..

[96] Amin H (2016). Par-4 dependent modulation of cellular beta-catenin by medicinal plant natural product derivative 3-azido Withaferin A. Mol. Carcinog..

[97] Suman S (2016). Oral administration of withaferin A inhibits carcinogenesis of prostate in TRAMP model. Oncotarget.

[98] Ioannou M (2018). Smad4 and epithelial-mesenchymal transition proteins in colorectal carcinoma: an immunohistochemical study. J. Mol. Histol..

[99] Hao, Y., Baker, D. & Ten Dijke, P. TGF-beta-mediated epithelial-mesenchymal transition and cancer metastasis. *Int. J. Mol. Sci.* **20**, 2767 (2019). 10.3390/ijms20112767 PMC6600375 31195692

[100] Pohl M (2010). SMAD4 mediates mesenchymal-epithelial reversion in SW480 colon carcinoma cells. Anticancer Res.

[101] Muller N (2002). Smad4 induces the tumor suppressor E-cadherin and P-cadherin in colon carcinoma cells. Oncogene.

[102] Reinacher-Schick A (2004). Loss of Smad4 correlates with loss of the invasion suppressor E-cadherin in advanced colorectal carcinomas. J. Pathol..

[103] Nayak D (2019). Indolylkojyl methane analogue IKM5 potentially inhibits invasion of breast cancer cells via attenuation of GRP78. Breast Cancer Res. Treat..

[104] Gonzalez DM, Medici D (2014). Signaling mechanisms of the epithelial-mesenchymal transition. Sci. Signal.

[105] Rah B (2012). A novel MMP-2 inhibitor 3-azidowithaferin A (3-azidoWA) abrogates cancer cell invasion and angiogenesis by modulating extracellular Par-4. PLoS ONE.

[106] Trotman LC, Pandolfi PP (2003). PTEN and p53: who will get the upper hand?. Cancer Cell.

[107] Azmi AS, Philip PA, Zafar SF, Sarkar FH, Mohammad RM (2010). PAR-4 as a possible new target for pancreatic cancer therapy. Expert Opin. Ther. Targets.

[108] Qiu G (1999). Mutually exclusive expression patterns of Bcl-2 and Par-4 in human prostate tumors consistent with down-regulation of Bcl-2 by Par-4. Oncogene.

[109] Furuse J (2018). An early clinical trial of Salirasib, an oral RAS inhibitor, in Japanese patients with relapsed/refractory solid tumors. Cancer Chemother. Pharm..

[110] Han Z, Liang J, Li Y, He J (2019). Drugs and clinical approaches targeting the antiapoptotic protein: a review. Biomed. Res. Int..

[111] Brown JS, Banerji U (2017). Maximising the potential of AKT inhibitors as anti-cancer treatments. Pharm. Ther..

[112] Sharma AK, Kline CL, Berg A, Amin S, Irby RB (2011). The Akt inhibitor ISC-4 activates prostate apoptosis response protein-4 and reduces colon tumor growth in a nude mouse model. Clin. Cancer Res..

[113] Azmi AS (2008). Critical role of prostate apoptosis response-4 in determining the sensitivity of pancreatic cancer cells to small-molecule inhibitor-induced apoptosis. Mol. Cancer Ther..

[114] Kline CL, Shanmugavelandy SS, Kester M, Irby RB (2009). Delivery of PAR-4 plasmid in vivo via nanoliposomes sensitizes colon tumor cells subcutaneously implanted into nude mice to 5-FU. Cancer Biol. Ther..

[115] Kim K (2019). Development of a novel prostate apoptosis response-4 (Par-4) protein entity with an extended duration of action for therapeutic treatment of cancer. Protein Eng. Des. Sel..

[116] Sarkar S (2015). Plant-derived SAC domain of PAR-4 (Prostate Apoptosis Response 4) exhibits growth inhibitory effects in prostate cancer cells. Front Plant Sci..

[117] Qin TJ (2010). Secretory expression of Par-4 SAC-HA2TAT following adeno-associated virus-mediated gene transfer induces apoptosis in HepG2 cells. Mol. Med. Rep..

[118] Zhang J, Sun A, Dong Y, Wei D (2018). Recombinant production and characterization of SAC, the core domain of Par-4, by SUMO fusion system. Appl. Biochem. Biotechnol..

[119] Irby RB, Kline CL (2013). Par-4 as a potential target for cancer therapy. Expert Opin. Ther. Targets.

[120] Wang P (2018). Neoadjuvant administration of hydroxychloroquine in a phase 1 clinical trial induced plasma Par-4 levels and apoptosis in diverse tumors. Genes Cancer.

[121] Guo Q, Xie J (2004). AATF inhibits aberrant production of amyloid beta peptide 1-42 by interacting directly with Par-4. J. Biol. Chem..

[122] Boosen M, Vetterkind S, Koplin A, Illenberger S, Preuss U (2005). Par-4-mediated recruitment of amida to the actin cytoskeleton leads to the induction of apoptosis. Exp. Cell Res.

[123] Park SK (2005). Par-4 links dopamine signaling and depression. Cell.

[124] Page G, Kogel D, Rangnekar V, Scheidtmann KH (1999). Interaction partners of Dlk/ZIP kinase: co-expression of Dlk/ZIP kinase and Par-4 results in cytoplasmic retention and apoptosis. Oncogene.

[125] Goswami A, Ranganathan P, Rangnekar VM (2006). The phosphoinositide 3-kinase/Akt1/Par-4 axis: a cancer-selective therapeutic target. Cancer Res..

[126] Roussigne M, Cayrol C, Clouaire T, Amalric F, Girard JP (2003). THAP1 is a nuclear proapoptotic factor that links prostate-apoptosis-response-4 (Par-4) to PML nuclear bodies. Oncogene.

[127] Goswami A (2008). Par-4 binds to topoisomerase 1 and attenuates its DNA relaxation activity. Cancer Res..

[128] Nguyen JQ, Irby RB (2017). TRIM21 is a novel regulator of Par-4 in colon and pancreatic cancer cells. Cancer Biol. Ther..

[129] Johnstone RW (1996). A novel repressor, par-4, modulates transcription and growth suppression functions of the Wilms’ tumor suppressor WT1. Mol. Cell Biol..

[130] Cheema SK (2003). Par-4 transcriptionally regulates Bcl-2 through a WT1-binding site on the bcl-2 promoter. J. Biol. Chem..

[131] Zhang Z, DuBois RN (2000). Par-4, a proapoptotic gene, is regulated by NSAIDs in human colon carcinoma cells. Gastroenterology.

[132] MacLean MA (2011). North American cranberry (Vaccinium macrocarpon) stimulates apoptotic pathways in DU145 human prostate cancer cells in vitro. Nutr. Cancer.

[133] Pereira MC (2013). Prostate apoptosis response-4 is involved in the apoptosis response to docetaxel in MCF-7 breast cancer cells. Int J. Oncol..

[134] Chow KU, Nowak D, Hofmann W, Schneider B, Hofmann WK (2005). Imatinib induces apoptosis in CLL lymphocytes with high expression of Par-4. Leukemia.

[135] Alvarez JV (2013). Par-4 downregulation promotes breast cancer recurrence by preventing multinucleation following targeted therapy. Cancer Cell.

[136] Huang YT, Chueh SC, Teng CM, Guh JH (2004). Investigation of ouabain-induced anticancer effect in human androgen-independent prostate cancer PC-3 cells. Biochem Pharm..

[137] Brasseur K (2015). Parasporin-2 from a new bacillus thuringiensis 4R2 strain induces caspases activation and apoptosis in human cancer cells. PLoS ONE.

[138] Rossi V (2011). Raloxifene induces cell death and inhibits proliferation through multiple signaling pathways in prostate cancer cells expressing different levels of estrogen receptor alpha and beta. J. Cell Physiol..

